# MicroRNA-based signatures impacting clinical course and biology of ovarian cancer: a miRNOmics study

**DOI:** 10.1186/s40364-021-00289-6

**Published:** 2021-07-13

**Authors:** E. Krasniqi, A. Sacconi, D. Marinelli, L. Pizzuti, M. Mazzotta, D. Sergi, E. Capomolla, S. Donzelli, M. Carosi, A. Bagnato, T. Gamucci, S. Tomao, C. Natoli, P. Marchetti, A. Grassadonia, N. Tinari, M. De Tursi, E. Vizza, G. Ciliberto, L. Landi, F. Cappuzzo, M. Barba, G. Blandino, P. Vici

**Affiliations:** 1 Division of Medical Oncology 2, IRCCS Regina Elena National Cancer Institute, Via Elio Chianesi 53, 00144 Rome, Italy; 2 UOSD Clinical Trial Center, Biostatistics and Bioinformatics, IRCCS Regina Elena National Cancer Institute, Via Elio Chianesi 53, 00144 Rome, Italy; 3 Department of Clinical and Molecular Medicine, Sant’Andrea Hospital, Medical Oncology Unit, Sapienza University, Via di Grottarossa 1035/1039, 00189 Rome, Italy; 4 Oncogenomic and Epigenetic Unit, IRCCS Regina Elena National Cancer Institute, Via Elio Chianesi 53, 00144 Rome, Italy; 5 Pathology Department IRCCS Regina Elena National Cancer Institute, Via Elio Chianesi 53, 00144 Rome, Italy; 6 Preclinical Models and New Therapeutic Agents Unit, IRCCS Regina Elena National Cancer Institute, Via Elio Chianesi 53, 00144, Rome, Italy; 7 Medical Oncology, Sandro Pertini Hospital, Via dei Monti Tiburtini 385, 00157 Rome, Italy; 8 Department of Radiological Oncological and Pathological Sciences, Division of Medical Oncology A, Sapienza University of Rome, Piazzale Aldo Moro 5, 00185 Rome, Italy; 9 Department of Medical, Oral & Biotechnological Sciences, University G. D’Annunzio, Via dei Vestini, 31, 66100 Chieti, Italy; 10 Department of Oncological Surgery, Gynecologic Oncologic Unit, IRCCS Regina Elena National Cancer Institute, Via Elio Chianesi 53, 00144 Rome, Italy; 11 Scientific Direction, IRCCS Regina Elena National Cancer Institute, Via Elio Chianesi 53, 00144 Rome, Italy

**Keywords:** Ovarian cancer, miRNAs, Prognostic/predictive biomarkers

## Abstract

**Background:**

In Western countries, ovarian cancer (OC) still represents the leading cause of gynecological cancer-related deaths, despite the remarkable gains in therapeutical options. Novel biomarkers of early diagnosis, prognosis definition and prediction of treatment outcomes are of pivotal importance. Prior studies have shown the potentials of micro-ribonucleic acids (miRNAs) as biomarkers for OC and other cancers.

**Methods:**

We focused on the prognostic and/or predictive potential of miRNAs in OC by conducting a comprehensive array profiling of miRNA expression levels in ovarian tissue samples from 17 non-neoplastic controls, and 60 tumor samples from OC patients treated at the Regina Elena National Cancer Institute (IRE). A set of 54 miRNAs with differential expression in tumor versus normal samples (T/N-deregulated) was identified in the IRE cohort and validated against data from the Cancer Genoma Atlas (TCGA) related to 563 OC patients and 8 non-neoplastic controls. The prognostic/predictive role of the selected 54 biomarkers was tested in reference to survival endpoints and platinum resistance (P-res).

**Results:**

In the IRE cohort, downregulation of the 2 miRNA-signature including miR-99a-5p and miR-320a held a negative prognostic relevance, while upregulation of miR-224-5p was predictive of less favorable event free survival (EFS) and P-res. Data from the TCGA showed that downregulation of 5 miRNAs, i.e., miR-150, miR-30d, miR-342, miR-424, and miR-502, was associated with more favorable EFS and overall survival outcomes, while miR-200a upregulation was predictive of P-res. The 9 miRNAs globally identified were all included into a single biologic signature, which was tested in enrichment analysis using predicted/validated miRNA target genes, followed by network representation of the miRNA-mRNA interactions**.**

**Conclusions:**

Specific dysregulated microRNA sets in tumor tissue showed predictive/prognostic value in OC, and resulted in a promising biological signature for this disease.

**Supplementary Information:**

The online version contains supplementary material available at 10.1186/s40364-021-00289-6.

## Introduction

In Western countries, ovarian carcinoma (OC) is the third most frequent gynecologic malignancy, and the leading cause of gynecological cancer-related death [1]. Epithelial OC is the most common hystologic subtype, accounting for more than 90% of cases [2]. The heterogeneous nature of the disease, along with the paucity of symptoms in the early phase, translates into a late diagnosis for approximately two-thirds of patients [3]. In advanced-stage tumors, patients often present with diffuse peritoneal spread, making radical surgery not feasible [4]. Cytoreductive surgery and chemotherapy combined with biological agents represent the standard of care for a relevant fraction of these patients. Usually, debulking surgery for advanced OC is followed by platinum-based chemotherapy, with disease relapse/progression occurring within 6 months in approximately 25% of patients. This latter percent estimate also defines the fraction of patients with platinum-resistant (P-res) tumors [4, 5]. Even though the remaining patients respond to initial chemotherapy, most of them will experience recurrence within 2–3 years due to acquired drug resistance [6]. Recently, surgical techniques have significantly improved and new targeted drugs such as poly ADP ribose polymerase (PARP)-inhibitors have been introduced in clinical practice [7]. However, the onset of drug resistance still translates into disappointing 5-year overall survival (OS) rates, which for all the stages combined are set at about 45% [8]. In such a scenario, biomarkers for OC early diagnosis, prognosis definition and prediction of treatment outcomes are eagerly needed. Combination of biomarkers such as Ca125, human epididymis protein 4 (HE4), and risk of ovarian malignancy algorithm (ROMA) index has been associated with increased performance compared to the use of single biomarkers [9]. Still, the unraveling of the biological mechanisms underlying the disease may significantly contribute to the identification and validation of adjunctive, increasingly accurate biomarkers, which may better orient the diagnostic workup and inform therapeutic decisions. A wide spectrum of biological processes involved in OC initiation and progression, including the potential of cancer cells to acquire invasive and metastatic properties as well as to develop drug resistance, are strongly impactedby epithelial-mesenchymal transition (EMT), a process whereby epithelial cells acquire a mesenchymal phenotype. The main regulators of EMT include signaling pathways suchs as WNT, and PI3K-AKT, and several transcription factors [10]. Relapse and resistance/sensitivity to platinum-compounds are also affected by biological mechanisms relatated to DNA damage and repair [11]. Moreover, cancer stem cells (CSC), a rare subset among the cancer population cells with stem cell features, are crucial for both cancer metastatization and chemoresistance [12].

The regulation of gene expression is crucial to for the vast majority of the aforementioned processes. The regulatory mechanisms act at the epigenetic, genetic, transcriptional, post-trascriptional, and translational level, with a wide spectrum of biological elements being involved at one or more of the prespecified contexes. MicroRNAs (miRNAs) consist of evolutionarily well conserved small non-coding RNAs of 19 to 23 nucleotides in length, which regulate gene expression by complementary base-pairing to the 3 ′untranslated region (UTR) of target mRNA, with consequent transcription inhibition, or direct degradation of target mRNA [13]. Hence, miRNAs act mainly via suppressing gene expression, and regulate approximately 30% of genes in the human genome [14]. The role of miRNA expression has been thoroughly investigated in cancers, including OC [15]. In particular, studies in OC have shown that miRNAs have a relevant impact on chemoresistance, metastatic pontetials, EMT and CSCs regulation [16, 17]. However, to our knowledge, a restricted number of investigators have performed a wide miRNA profiling in OC aimed at the identification/validation of miRNA expression biological fingerprints that extend beyond the prognostic and/or predictive relevance.

We herein present results from an observational study based on tissue miRNA profiling performed in a cohort of OC patients and matched controls from the Regina Elena National Cancer Institute (IRE). MicroRNAs identified in neoplastic and non-neoplastic tissues were tested for differential expression, and for prognostic/predictive value, with the attempt to validate results against data from the Cancer Genome Atlas (TCGA) and other pertinent public datasets. Relevant miRNAs were evaluated as a biological signature of OC, enrichment analysis was performed using their relative target genes, and finally a network representation of the miRNA-mRNA interactions was carried out. Results were finally interpreted and discussed in light of the most recent inherent literature.

## Materials and methods

### Case selection and definition of relevant clinical outcomes

This is a single institution study that retrospectively identified patients with an OC, treated at the IRE. For the purposes of this study, non-neoplastic ovarian tissue samples stored at the IRE biobank served as controls. The study protocol was approved by the IRE Ethics Committee. Our study was carried out in agreement with the Declaration of Helsinki and adheres to the Reporting Recommendations for Tumor Marker Prognostic Studies guidelines.

This study included patients treated with a platinum-based regimen administered as neo-/adjuvant/first-line treatment in the early or advanced setting for histologically-confirmed OC. Additional inclusion criteria were age ≥ 18 years and baseline ECOG performance status 0–1. Patients were excluded if they had a diagnosis of a second tumor. Patients treated with a platinum-based regimen in the adjuvant setting were included if they had received at least 6 cycles, while patients treated in the neoadjuvant setting were included if having received at least 3 cycles of treatment before surgery, and completed at least 6 cycles following surgery. Patients treated in the metastatic setting or any other patient with persistent/recurrent disease after surgery could have also been treated with bevacizumab added to chemotherapy and as a subsequent maintenance therapy, according to the international guidelines.

Non-neoplasic ovarian tissue samples from 17 age-matched women who underwent abdominal surgery at the IRE served as controls. In this group of study participants, the oophorectomy was concomitant to hysterectomy and motivated by uterus fibromatosis complicated by or at high risk of hemorrhagic events. The primary aim of this study was to identify biomarkers of prognostic and/or predictive relevance throughout miRNA expression profiling in tumoral tissues. Validation was planned using the TCGA data. To our study purposes, the following endpoints were chosen: overall survival (OS), event free survival (EFS) and platinum-sensitivity status (PSS). Overall survival was defined as the time elapsed from the date of the first cycle of neo-/adjuvant/first-line chemotherapy to the date of death from any cause. Event free survival was measured in months, and was calculated according to the disease setting. For patients with *ab initio* metastatic disease, who received a first-line treatment, EFS was calculated from the date of the first chemotherapy to disease progression or death from any cause. For patients who received chemotherapy in the neo−/adjuvant setting, EFS was calculated as the time from the first cycle of chemotherapy to disease relapse/recurrence or death from any cause. Platinum sensitivity status was categorized on the basis of time in months from the last cycle of platinum-based chemotherapy to disease progression/relapse/recurrence. When this time interval was ≤6 months, the tumor was classified as platinum-resistant (P-res), otherwise, it was considered platinum-sensitive (P-sens).

### MicroRNA data extraction for the IRE cohort

Signals from miRNAs arrays were verified for quality control and extracted by Agilent Feature Extraction 10.7.3.1 software. The arrays were quantile normalized. All values lower than 1 were considered below detection and thresholded to 1 and data were log2-trasformed. MiRNAs not expressed in at least 50% of the samples were excluded.

A second analytical method consisting in real-time polymerase chain reaction (RT-PCR) was performed in 10 ovarian tumor and 10 normal tissue samples from the IRE cohort, to quantify the expression level of 3 representative miRNAs.

### MicroRNA data extraction from the TCGA cohort

Normalized miRNA and gene expression profiles of high-grade serous OC (HG-SOC) were obtained from Broad Institute TCGA Genome Data Analysis Center (2016): TCGA data from Broad GDAC Firehose 2016_01_28 run. Broad Institute of MIT and Harvard. Dataset. (10.7908/C11G0KM9) (http://gdac.broadinstitute.org/runs/stddata_2016_01_28/data/OV/20160128/).

MiRNA expression in the TCGA cohort was performed by both miRNA sequencing and miRNA array profiling. MicroRNA sequencing data were used for validating predictive/prognostic miRNAs, while miRNA array data were used for validating tumor versus normal deregulation (only this second dataset provided miRNA expression data on normal ovarian tissue samples).

### MicroRNA data extraction from GEO and GTEx

MiRNA expression data of ovarian cancer tissue and normal tissue were extracted from the GEO database with accession number GSE119055 (6 ovarian cancer tissue samples and 3 normal ovarian tissue samples run on Affymetrix miRNA array), and GSE83693 (8 primary ovarian cancer tissue and 4 normal ovarian tissue on Agilent platform). Data were analyzed by GEO2R software.

MiRNA expression data of 88 normal ovarian tissue samples was obtained from GTEx dataset and combined with miRNA expression data of tumor samples from TCGA RNAseq dataset using GEPIA2 web tool (http://gepia2.cancer-pku.cn/#index).

### Gene set enrichment analysis, target prediction/validation, and network analysis

A Preranked Gene Set Enrichment Analysis (GSEA) (https://www.gsea-msigdb.org/gsea/index.jsp) was performed on a list of genes correlated to a selected groups of 9 miRNAs. The GSEA algorithm calculates an enrichment score reflecting the degree to which the genes included in a gene set are overrepresented at the top or bottom of the ranked list of all genes present in the expression dataset. Correlation was evaluated on matched miRNA\mRNA samples of TCGA casuistry based on the Spearman’s correlation coefficient.

The gene list for enrichment analysis was restricted to those genes that resulted 1) differentially expressed between 88 normal samples of the GTEx dataset and 499 ovarian tumor samples from the TCGA, and 2) coherently modulated according to an anti-correlating fashion with respect to the tumor versus normal deregulation of the relative miRNAs.

Gene Set Enrichment Analysis was run in preranked mode using classic as metric and 1000 permutations selecting the curated gene sets of Molecular Signatures Database (MsigDB) derived from Hallmark, Kyoto Encyclopedia of Genes and Genomes **(**KEGG) and oncogenic collections. Gene sets enrichment was assessed by positive and negative normalized enrichment score (NES).

MicroRNA\mRNA predicted interactions and enrichment analysis were performed by miRWalk version 3 (http://mirwalk.umm.uni-heidelberg.de/search_mirnas/) and miRDB (http://mirdb.org/index.html).

A miRNA-centric network based on validated target of the miRNA signature was created with miRNet (https://www.mirnet.ca/miRNet/home.xhtml). MiRNA target gene data were collected from well-annotated databases: miRTarBase v8.0, TarBase v8.0 and miRecords.

We used the same targets genes as for GSEA to also build a Protein-Protein Interaction Network by STRING database (ShinyGO v0.61).

### Statistical analysis

Descriptive statistics were used to calculate frequencies and represent distributions of patient-, tumor- and treatment-related features. Overall survival and EFS were evaluated by Kaplan-Meier method and the log-rank test was used to establish the statistical significance of the distance between medians and/or curves. The impact of clinical variables on the survival curves was investigated by univariate Cox proportional hazard regression models. High and low levels of specific features were assessed by positive and negative z-score, respectively. MicroRNA signature level was defined by considering the average z-scores of the selected miRNAs.

Differences between subgroups of samples were evaluated applying unpaired T-test, Wilcoxon test, ANOVA test, Chi square test or logistic regression according to the specific data distributions and number of subsets compared. Tukey post-hoc test was employed when appropriate. A false discovery procedure was included for multiple comparisons. Statistical significance was set at 5%.

Unsupervised hierarchical clustering analysis (HCA) was performed to individuate specific patterns of expression using the Euclidean distance metric. After normalization, miRNA expression data was used to perform eigen-vector-based multivariate analysis by principal component analysis (PCA). Differential miRNA expression data was displayed using volcano plots. Analyses were performed by using MATLAB R2019b software.

**Fig. 1:**
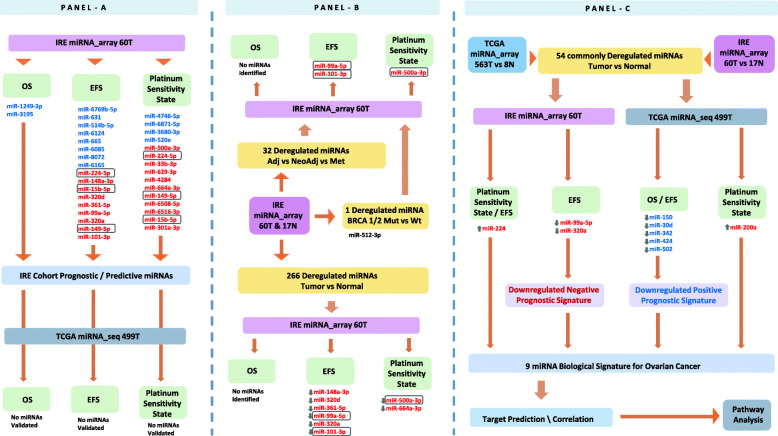
Workflow of the study. Identification of prognostic/predictive miRNAs in the Regina Elena National Cancer Institute cohort (IRE miRNA_array 60 T), and validation in the TCGA cohort (TCGA miRNA_array 563 T, and TCGA miRNA_seq 499 T) (**a**). Differential miRNA expression analysis according to disease setting, BRCA 1/2 status, and in tumor versus normal tissue for the IRE cohort (**b**). TCGA validation of the tumor versus normal dysregulated miRNAs, and identification of prognostic and biological signatures based on their deregulation (**c**). Abbreviations: EFS = event free survival; OS = overall survival; T = tumor samples. MiRNAs included in the rectangular shape intersect within the same panel. MiRNAs in red color are negatively associated to prognosis and platinum sensitivity, while those in blue color are positively associated with the same outcomes. MiRNAs preceded by a upward arrow are upregulated in cancer tissue with respect to normal tissue, while those preceded by a downward arrow are downregulated

A summary workflow of the analysis that we conducted on the set of miRNAs detected in the IRE cohort, including the modalities of the external validations, miRNA signature selection, and pathway analysis, is represented in Fig. 1.

## Results

### IRE cohort characteristics and clinical outcomes

Sixty samples of OC and 17 non-neoplastic ovarian tissue samples were collected from patients treated at the IRE from 2007 through 2015. The median follow-up for the whole cohort was 47.5 months, with the last update of time-to-event clinical outcomes carried out in July 2020. The median age at diagnosis of OC patients was 55-years (range: 36–81). Six (10%) of these patients carried a germinal mutation in BRCA1 or BRCA2 genes. Regarding histology, 45 (75.0%) of them had a serous carcinoma, 4 (6.7%) had a clear cell carcinoma, 1 (1.6%) patient had a mixed serous and clear cell carcinoma, 6 (10.0%) had a poorly differentiated carcinoma, 4 (6.7%) had a not otherwise specified OC. All 60 patients started a systemic treatment with carboplatin plus paclitaxel in a neo−/adjuvant or metastatic setting. Two patients switched to cisplatin plus gemcitabine and carboplatin plus docetaxel from the second cycle, due to allergic reactions occurred following exposure to the initially administered regimen. The timing of systemic treatment administration of the primary platinum-based regimen was dependent on the clinical stage at diagnosis. Eleven (18.3%) patients presented with metastatic disease at the initial diagnosis (FIGO clinical stage IV), therefore, received the platinum-based regimen as a first-line treatment. Four (6.7%) of them also received bevacizumab in association with chemotherapy. Twenty-seven (45.0%) patients presented with an apparently operable disease (FIGO clinical stage I - IIIB), thus receiving surgery as an up-front treatment and platinum-based chemotherapy in the adjuvant setting. Twenty-two (36.7%) patients presented at the initial diagnosis with a non-metastatic, non-operable disease (FIGO clinical stage IIIC), and were treated with an up-front platinum-based neoadjuvantchemotherapy. All these patients underwent surgery following neodjuvant treatment. Main patient-and disease-related characteristics for the IRE cohort are summarized in Table 1.

**Table 1:** Summarized characteristics of IRE cohort patients (*N* = 60)

Characteristics	N(%)
Age yr, median (range)	55 (36–81)
BRCA 1/2 status	
*Wild Type*	54 (90%)
*Mutated*	6 (10%)
Hystology	
*Serous carcinoma*	45 (75.0%)
*Clear cell carcinoma*	4 (6.7%)
*Mixed serous and clear cell carcinoma*	1 (1.6%)
*Poorly differentiated carcinoma*	6 (10.0%)
*Not otherwise specified carcinoma*	4 (6.7%)
Disease setting (FIGO Stage)	
*Metastatic (Stage IV)*	11 (18.3%)
*Adjuvant (Stage I - IIIB)*	27 (45.0%)
*Neoadjuvant (IIIC)*	22 (36.7%)
First systemic treatment	
*Carboplatin + Paclitaxel*	54 (90%)
*Carboplatin + Paclitaxel + Bevacizumab*	4 (6.8%)
*Carboplatin + Docetaxel*	1 (1.6%)
*Cisplatin + Gemcitabine*	1 (1.6%)

The median EFS was 21.9 months (mo) (range: 1.1–147.5) for the whole IRE OC patient population. The median EFS was 14.1 mo (range: 1.1–37.2), 15.5 mo (range: 4.9–89.3) and 38.0 mo (range: 3.7–147.5) for patients treated in the metastatic, neoadjuvant and adjuvant setting, respectively (log-rank *p* = 0.001). The median OS was 56.5 mo (range: 3.4–147.5) for the whole cohort. Median OS was 41.9 mo (range: 3.4–71.8), 52.8 mo (range: 14.3–108.9) and 105.7 (range: 3.7–147.5) mo for patients treated in the metastatic, neoadjuvant and adjuvant setting, respectively (log rank *p* = 0.002). Regarding PSS, 19 (31.7%) and 39 (65.0%) patients were respectively classified as having P-res and P-sens tumors. For 2 (3.3%) patients, platinum-sensivity could not be evaluated because point censoring occurred before 6 months with respect to the last follow-up. Patients with metastatic disease or having received neodjuvant therapy tended to have more frequently a P-res disease compared to patients treated with adjuvant systemic therapy (Chi square test, *p* = 0.022).

### Correlation between differential miRNA expression and clinical outcomes in the IRE cohort

Microarray-based miRNA expression profiling was carried out in tumor samples of 60 patients diagnosed with OC and in non-neoplastic tissue samples from 17 women. Only miRNAs with an AUC > 0.9 were included in further analysis. We started our analysis by searching for expressed miRNAs with a relevant impact on the prespecified clinical endpointsin the IRE cohort (Fig. 1, Panel a).

**Fig. 2:**
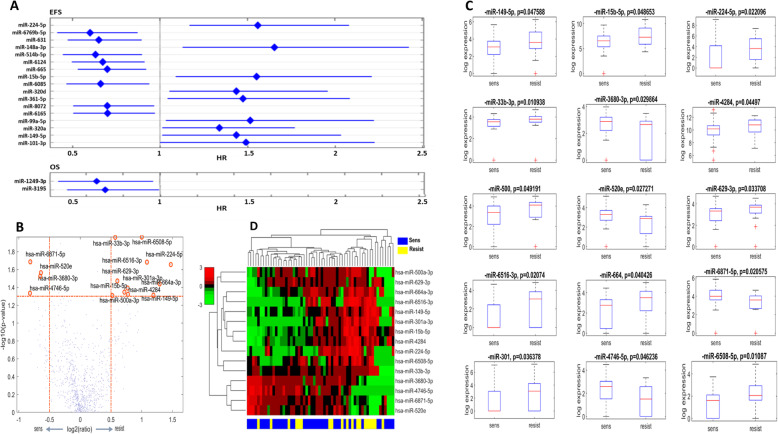
Identification of prognostic and predictive miRNAs in the IRE cohort. Forest plots indicating miRNAs with statistically significant (*p* < 0.05) impact on EFS and OS in the IRE cohort (**a**). Volcano plot showing the differentially expressed miRNAs between 39 platinum sensitive and 19 platinum resistant tumor samples (**b**). Box plots displaying miRNAs with statistically significant (Wilcoxon test, *p* < 0.05) differential expression between 39 platinum sensitive and 19 platinum resistant tumors (**c**). Heatmap illustrating the unsupervised hierarchical clustering analysis of the 15 miRNAs predictive of platinum sensitivity state (**d**), profiled on 39 platinum sensitive and 19 platinum resistant tumors. Abbreviations: HR = hazard ratio; sens = platinum sensitive; resist = platinum resistant

For the detection of miRNAs whose expression had an impact on EFS and OS, we employed univariate Cox regression and selected only those with a statistically significant effect. We identified 8 miRNAs whose expression correlated with longer EFS, i.e., miR-6769b-5p, miR-631, miR-514b-5p, miR-6124, miR-665, miR-6085, miR-8072, miR-6165. Conversely, 9 miRNAs impacted EFS negatively, i.e., miR-224-5p, miR-148a-3p, miR-15b-5p, miR-320d, miR-361-5p, miR-99a-5p, miR-320a, miR-149-5p, and miR-101-3p (Fig. 2 Panel a). Regarding OS, we identified 2 miRNAs associated with a statistically significant impact for lower risk of death, i.e., miR-1249-3p, miR-3195 (Fig. 2 Panel a).

Differential miRNA expression analysis between 19 P-res and 39 P-sens tumors allowed to identify 15 miRNAs with statistically significant deregulation (Fig. 2, Panel b). Four of them, namely, miR-4746-5p, miR-6871-5p, miR-3680-3p and miR-520e, resulted significantly more expressed in P-sens tumors compared to their resistant counterpart, while miR-500a-3p, miR-33b-3p, miR-629-3p, miR-4284, miR-664a-3p, miR-149-5p, miR-6508-5p, miR-6516-3p, miR-15b-5p, miR-301a-3p and miR-224-5p were significantly more expressed in P-res tumors (Fig. 2, Panel c). Even though each of the 15 miRNAs correlated with PSS, when we performed hierarchical clustering analysis (HCA) on the 60 IRE tumor samples including all the 15 miRNAs, no separation between P-res and P-sens samples emerged (Fig. 2, Panel d).

Noteworthy, among the miRNAs predictive of shorter EFS, miR-224-5p, miR-15b-3p, and miR-149-5p were in common with the miRNAs predictive of P-res (Fig. 1, and Fig. 2).

For validation purposes, we used TCGA data obtained throughout RNA sequencing of 499 tumor samples. None of the prognostic/predictive miRNAs identified based on the analysis performed in the IRE cohort was validated in the TCGA cohort (Fig. 1, Panel a).

### Differential miRNA expression in the IRE cohort according to disease setting and BRCA 1/2 status

In the IRE cohort, 32 miRNAs with a statistically significant differential expression by disease setting were identified (Supplementary Table 1). Among them, only 3 had emerged in prior analysis. In more detail, miR-99a-5p and miR-101-3p were significantly deregulated in patients treated within each clinical setting. These same miRNAs were also among those correlated with shorter EFS (Fig. 2, Panel a). A two-by-two groups comparison using Tukey post-hoc test showed that these two miRNAs were more expressed in tumor samples of patients treated in the neoadjuvant setting with respect to those treated in the adjuvant setting (respectively, *p* = 0.007 and *p* = 0.010). The third miRNA was miR-500a-3p, which we previously found to be more expressed in tumor samples of patients with P-res disease, compared to those with P-sens disease (Fig. 2, Panel c). This miRNA resulted also significantly more expressed in tumor samples from patients in the neoadjuvant setting compared to those in the adjuvant setting (post-hot Tukey test *p* = 0.034).

Differential miRNA expression analysis between the 6 patients BRCA 1/2 mutated and their wild type counterpart detected only 1 miRNA with a significant deregulation. In particular, miR-512-3p resulted less expressed in the tumor samples of BRCA 1/2 mutated patients, when compared to the wild type ones (Wilcoxon test *p* = 0.040). This miRNA was not found among those impacting clinical outcomes in the IRE cohort, nor among miRNAs deregulated according to the specific clinical setting. A summary of the analyzes described in this section is represented in Fig. 1, Panel b.

### Differential miRNA expression between tumor and normal tissue in the IRE cohort

**Fig. 3:**
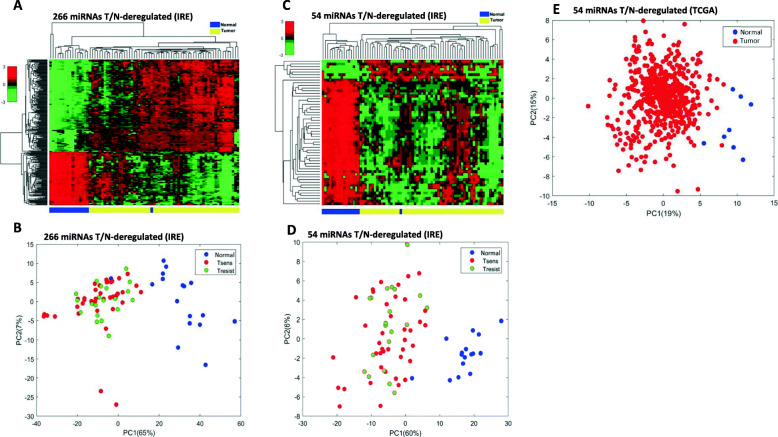
Dimensionality reduction analysis of differentially expressed miRNAs between tumor samples and normal. Unsupervised hierarchical clustering analysis (**a**) and principal component analysis (**b**) using the expression levels of the 266 miRNAs differentially expressed in tumor tissue versus normal tissue in the Regina Elena National Cancer Institute (IRE) cohort. These miRNAs separate the two types of samples, i.e., tumoral and normal tissue samples. This was confirmed by hierarchical clustering analysis in the IRE cohort (**c**), and by principal component analysis in the IRE cohort (**d**), and TCGA cohort (**e**), when using the 54 miRNAs validated against TCGA data. Statistical differences in miRNAs expression were assessed by permutation test and Student’t T-test. Abbreviations: T/N = tumor versus normal; Tsens = platinum sensitive tumor; Tresist = platinum resistant tumor; PC1 = first principal component; PC2 = second principal component; TCGA = The Cancer Genome Atlas

Differential expression analysis between tumor and normal tissue (T/N) showed 266 miRNAs having a statistically different expression level between the two sample sets (Supplementary Table 2). A HCA using the expression level of these 266 miRNAs showed a separation between tumor samples and normal tissue samples (Fig. 3, Panel a). Subsequent principal component analysis (PCA) was performed to quantify the miRNA expression variation between tumor and normal tissue samples and identify possible clusters. Tumor samples were also labeled separately in accordance to their PSS. As shown in Fig. 3, panel b, there was a 65.0% variation in the first principal component and a 7.0% variation in the second principal component, which translated into a clear-cut separation between tumor and normal tissue samples. Conversely, no separation emerged among P-sens and P-res samples. Both, the supervised HCA and PCA, showed the normal tissue from only 1 individual (with ID 80) clustering better with the tumor tissue samples than other normal samples (Fig. 3, panels a and b).

None of the two miRNAs impacting OS in the IRE cohort could be found in the tumor versus normal deregulated (T/N-deregulated) miRNA set. Conversely, 6 among the miRNAs found to be downregulated in the tumor samples with respect to normal tissue (T/N-downregulated), namely miR-148a-3p, miR-320d, miR-361-5p, miR-99a-5p, miR-320a and miR-101-3p (T-test *p*-values for all of them< 0.001), were also listed among the miRNAs that were previously detected as predictive of shorter EFS in the IRE cohort (Fig. 2, Panel a). Moreover, as described in the previous section, miR-99a-5p and miR-101-3p were also found to be more expressed in tumor samples of patients treated in the neoadjuvant setting, with respect to those treated in the adjuvant setting. Among the T/N-deregulated miRNAs, 2 miRNAs coincided with miRNAs predictive of PSS. Specifically, miR-500a-3p and miR-664a-3p, which resulted enriched in P-res tumor samples in the previous analysis (Fig. 2, Panel c), were also found dowregulated in the tumor samples, with respect to normal tissue (T-test p-value for both < 0.001). Interestingly, miR-500a-5p was also found to be T/N-upregulated in patients treated in the neodjuvant setting, when compared to those treated in the adjuvant setting. A summary of the analyzes described in this section is represented in Fig. 1, Panel b.

### External validation of IRE cohort T/N-deregulated miRNAs

To validate T/N-deregulated miRNAs detected in the IRE cohort, we used data obtained by array profiling of 8 normal tissue samples and 563 tumor samples of the same TCGA cohort. Among the 266 miRNAs that were T/N-deregulated in the IRE cohort, 54 were concordantly and significantlyT/N-deregulated also in the TCGA cohort (Supplementary Table 3). Hence, 20.3% of T/N-deregulated miRNAs in the IRE cohort were validated against data from the TCGA cohort. Hierarchical clustering analysis and PCA were performed using exclusively the expression levels of the 54 T/N-deregulated miRNAs identified in the IRE cohort and validated in TCGA cohort. The HCA confirmed the clear separation between Tumor and Normal tissues also in this case (Fig. 3, Panel c). Regarding PCA, 60% of the variation was found in the first principal component and 6% in the second, which is consistentwith the results from the PCA in the IRE cohort (Fig. 3, Panel d). Also in this case, no separation according to PSS was observed. Moreover, the same normal tissue sample falling among tumor samples (ID 80) outlied again the clustering from normal samples by classifying into the group of tumor samples in both HCA and PCA. The separation by PCA of tumor samples from normal samples using the 54 commonly T/N-deregulated was also validated in the TCGA cohort, with first and second principal components’ variations of respectively 20 and 16% (Fig. 3, Panel e).

To further consolidate the validation of the IRE cohort’s miRNA T/N deregulation against the TCGA data, we performed an additional experiment by using a different analytic technique represented by real time polymerase chain reaction (RT-PCR). In more detail, we randomly selected from the IRE cohort a subgroup including 10 samples of ovarian tumor tissue and 10 samples of normal tissue. Then RT-PCR was performed on these samples to measure the expression of level of 3 representative miRNAs chosen among the 54 commonly T/N deregulated with respect to TCGA. All these three miRNAs were confirmed to have the same T/N-deregulation as was found by array profiling in the IRE cohort and confirmed common deregulation with respect to TCGA. Specifically, miR-99a-5p and miR-145-5p resulted T/N-downregulated, and miR-224-5p T/N-upregulated (Supplementary Fig. 1, panels A, B and C).

Moreover, by taking into account the low number of only 8 normal ovarian tissue samples in the TCGA cohort, we performed a confirmatory analysis on the 54 commonly T/N-deregulated miRNAs by using data from 2 additional studies. Data from the GEO database were used with accession number GSE119055, which includes 6 OC tissue samples and 3 normal ovarian tissue samples, run on Affymetrix miRNA array, and GSE83693, which includes 8 primary OC tissue samples and 4 normal ovarian tissue, run on Agilent platform. We respect to our 54 T\N deregulated miRNAs, we found correspondence for 44 miRNAs in the first dataset and 40 miRNAs in the second dataset. Around 80% of the miRNAs showed the same modulation, confirming our results (Supplementary Table 4).

Noteworthy, none of the miRNAs impacting OS or predictive of PSS in the IRE cohort were found among the 54 commonly T/N-deregulated IRE-TCGA miRNAs. However, two miRNAs impacting EFS negatively in the IRE cohort were present among the 54 validated miRNAs. Specifically, miR-99a-5p and miR-320a were both related to shorter EFS in the IRE cohort and resulted dowregulated in tumor samples with respect to normal tissue, in both the IRE and the TCGA cohorts (Fig. 1, Panel c; and Fig. 2 Panel a).

### Characterization of prognostic/predictive miRNA signatures based on T/N-deregulation

For the characterization of viable miRNA signatures impacting the clinical course and/or the biological aspects of OC we prioritized selection from the 54 miRNAs that were T/N-deregulated in the IRE cohort, and that had such deregulation validated in the TCGA cohort. The secondary criteria for miRNA selection that was employed in the pursue of signatures, required the selected miRNAs to have significant impact on clinical outcomes in the IRE cohort and/or in the TCGA cohort (Fig. 1, Panel c).

**Fig. 4:**
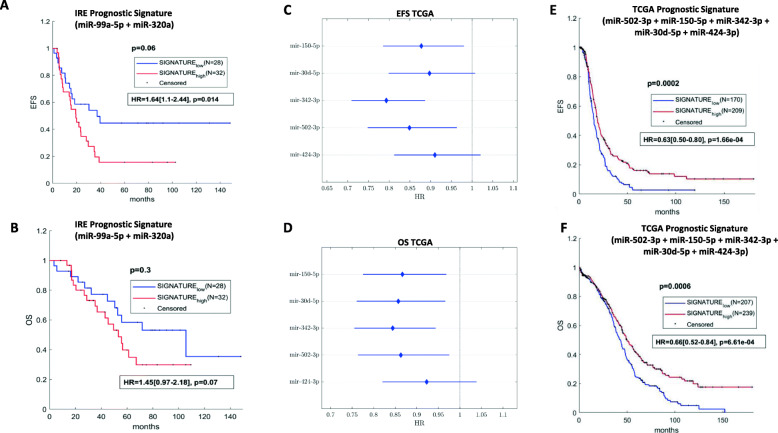
Characterization of miRNA prognostic signatures in the IRE and TCGA cohorts**.** Kaplan-Meier survival curves of event free survival (EFS) (**a**), and overall survival (OS) (**b**), of patients with low versus high expression of the 2-miRNA prognostic signature identified in the IRE cohort (statistically significant differences when logrank test *p* < 0.05). Forest plot illustrating univariate Cox regression analysis for EFS (**c**) and OS in the TCGA of the 5 miRNAs selected for the inherent prognostic signature (**d**). Kaplan-Meier survival curves of event free survival (EFS) (**e**), and overall survival (OS) (**f**), of patients with low versus high expression of the TCGA 5 miRNA prognostic signature (statistically significant differences when logrank test *p* < 0.05). Univariate Cox regression for prognostic signatures is displayed beneath the relative Kaplan-Meier curves, inside a rectangle. Abbreviations: HR = hazard ratio

We identified only 2 miRNAs profiled in the IRE cohort satisfying both criteria of selection by following this method. In particular, the 2 miRNAs were miR-99a-5p and miR-320a, which were already mentioned in the previous section asT/N-downregulated in the IRE and TCGA cohorts, and at the same time significantly associated to worse EFS in the IRE cohort. MiR-99a-5p was also relatively more expressed in the tumors of patients who had received a neoadjuvant treatment, when compared to those treated by adjuvant chemotherapy, in the IRE cohort. MiR-99a-5p, and miR-320a were unified into a single T/N-downregulated prognostic signature, which showed to negatively impact EFS (HR 1.64, 95%CI 1.10–2.44, *p* = 0.014), and OS (HR 1.45, 95%CI 0.97–2.18, *p* = 0.07) (Fig. 4, Panel a and Panel b), in the IRE cohort. However, this signature was not prognostic in the TCGA cohort (Supplementary Fig. 2, Panels A and B). Noteworthy, we identified a third miRNA at the limit of our criteria for the selection. Specifically, miR-224, which was found to be correlated with P-res and shorter EFS in the IRE cohort (Fig. 2, and Supplementary Fig. 2, Panel C), was also T/N-upregulated in both the TCGA cohort (Suplementary Fig. 2, Panel D) and the IRE cohort, not reaching statistical significance in the latter (Supplementary Fig. 2, Panel E). However, this miRNA resulted significantly T/N-upregulated when the subset analysis using RT-PCR was performed (Supplementary Fig. 1, Panel C). We decided to keep this miRNA as an optimizing factor for subsequent pathway analysis.

In a further analysis, the 54 miRNAs validated in the TCGA for T/N-deregulation, were tested for prognostic and predictive impact inthe TCGA cohort. Six miRNAs with an impact on clinical outcomes in the TCGA cohort were identified (Fig. 1, Panel c). In particular, miR-502-3p, miR-150-5p, miR-342-3p, miR-30d-5p, and miR-424-3p, which were T/N-dowregulated, had a positive prognostic effect for both OS and EFS in the TCGA cohort (Fig. 4, Panel c and Panel d). In supplementary Fig. 3 and Supplementary Fig. 4, Kaplan Meier curves for EFS and OS are displayed. Higher expression levels for each of the selected 5 miRNAs were associated with better outcomes for both the variables. We built a T/N-downregulated positive prognostic signature using these 5 miRNAs, which showed a statistically significant and coherent effect on both OS and EFS in the TCGA cohort (Fig. 4, Panel e and Panel f). The sixth miRNA, namely miR-200a-5p, was previously detected among the T/N-upregulated in both the IRE and TCGA cohorts, and impacted PSS in the TCGA cohort, resulting correlated with P-res (Supplementary Fig. 2, Panel F).

### Selection of a biological signature and its use for pathway analysis

Concerning the selection of a set of miRNAs to be structured as a biological signature adequate for gene ontology analysis, we used tha same inclusion criteria previously defined in terms of T/N-deregulation and impact on clinical outcomes in the IRE and TCGA cohorts. Hence, we included in this signature the 7 miRNAs from the 2 prognostic signatures identified in the previous section (miR-99a-5p and miR-320a from the IRE cohort, and miR-502-3p, miR-150-5p, miR-342-3p, miR-30d-5p, and miR-424-3p from the TCGA cohort), and the 2 miRNAs selected as predictive representatives (miR-224 from the IRE cohort and miR-200a-5p from the TCGA cohort).

The resulting biological signature was composed of 9 miRNAs. As illustrated in Supplementary Fig. 5, the 7 miRNAs impacting prognosis were T/N-downregulated in both the IRE cohort (Panel A and Panel B), and in the TCGA cohort (Panel C). Conversely, the 2 miRNAs selected as predictive of PSS, were upregulated in both datasets.

Owing to the fact that the main criterion for the 9 miRNA signature was the T/N-deregulation, we performed an ultimate confirmatory analysis on this aspect by using a combination of data from 88 normal samples from GTEx dataset and the tumor samples from TCGA RNA sequencing-based dataset. A PCA was carried out using differential T/N-expression of all the target genes negatively correlated to the 9 miRNA signature. The targets genes were able to clearly discriminate tumors from normal samples (Supplementary Fig. 6).

**Fig. 5:**
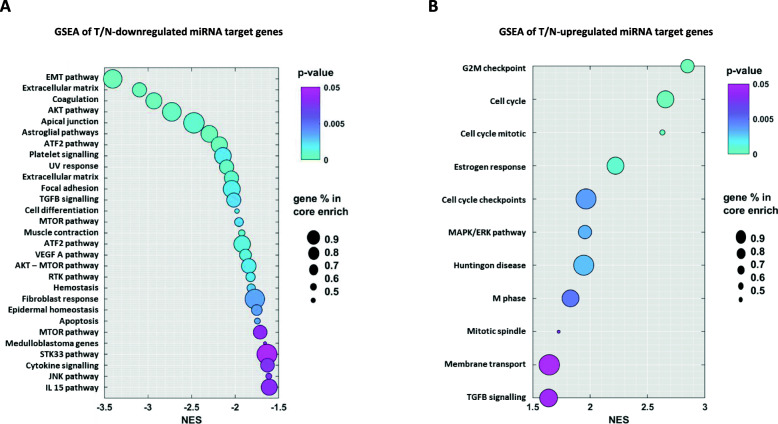
Gene enrichment analysis of the 9 miRNA-biological signature. Enrichment analysis of 7 T/N-downregulated (**a**), and 2 T/N-upregulated (**b**) miRNA gene targets. Analysis was performed by GSEA 4.1.0 software (BROAD Inst.) and run in pre-ranked mode. Predicted targets were ranked based on the correlation coefficients obtained from miRNA\mRNA TCGA RNA-sequencing data. Results showed normalized scores for negative enrichment (**a**) and positive enrichment (**b**). Color bar indicates the false discovery rate and the circle size represents the percentage of the genes in the core enrichment pathway. Abbreviations: T/N = tumor versus normal; NES = normalized enrichment score; path. = pathway; sign. = signaling

Once the 9 miRNA signature was set, we derived a relative target gene list to be used for a gene set enrichment analysis (GSEA) (Supplementary Table 5). Genes for this list were selected according the following criteria: i) anti-correlation to the 9 miRNAs of the signature based on target prediction using miRWalk and miRDB, and target validation using miRNA/mRNA expression data from the TCGA RNA sequencing-based dataset; ii) coherent T/N-deregulation with the 9 miRNAs of the signature based on the differential expression analysis of the selected genes between 88 normal samples of the GTEx dataset and the 499 ovarian tumor samples of the TCGA RNA sequencing-based dataset. After the gene list was defined, enrichment analysis was performed on immunogenic/oncogenic gene signatures and cancer hallmarks related sets of genes according to Kyoto Encyclopedia of Genes and Genomes (KEGG). The genes targeted by the 7 T/N-downregulated miRNAs, which should result presumably upregulated in the tumor with respect to normal tissue, showed the highest enrichment for oncogenic pathways such as EMT pathway, AKT signaling, and mTOR signaling, but also for ATF2 signaling, TGF-beta signaling, VEGF A signaling and other less specific cancer sustaining pathways (Fig. 5, Panel a). On the other hand, the most enriched terms in the GSEA of the genes negatively modulated by the 2 T/N-upregulated miRNAs were the tumor suppressing signaling pathways related to cell cycle regulating genes and MAPK/ERk pathway (Fig. 5, Panel b).

**Fig. 6:**
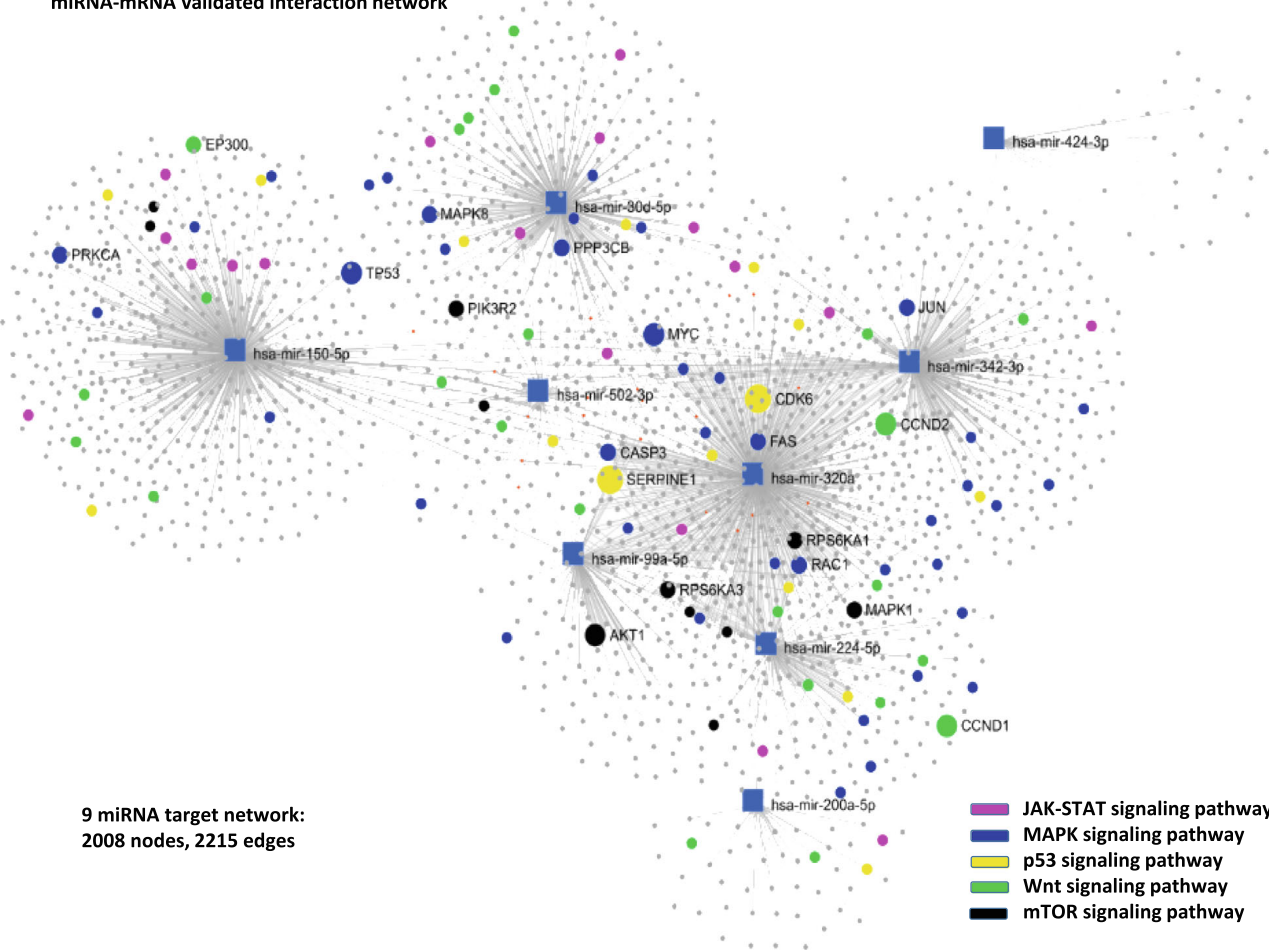
Network analysis of 9 signature miRNAs using validated miRNA-mRNA interactions. Signature miRNAs were applied to a miRNA-centric network visual analytics platform (MiRNet). miRNA target gene data were collected from well-annotated database: miRTarBase v8.0, TarBase v8.0 and miRecords. Targets were used to build a network of main interactions. Nodes indicated by rectangles represent signature miRNAs. Nodes indicated by circles represent target genes. A standard enrichment analysis based on the hypergeometric tests after adjustment for false discovery rate (FDR) was also included

Lastly, we conducted a network analysis relative to the 9 miRNAs of the biological signature by considering only validated miRNA-mRNA interactions from the TCGA RNA-sequencing based dataset. The interaction network resulted very dense with 2008 nodes and 2215 edges. For a selective visualization of the most important target genes in the network, we highlighted the interactions of the following relevant pathways: the JAK-STAT, MAPK, P53, Wnt and mTOR signaling pathway (Fig. 6). A complete list of the validated target genes is represented in supplementary Table 6, and the pathways detected by the target network analysis are displayed in supplementary Table 7. As illustrated in Fig. 6, the most intensively regulated genes by our 9 miRNA biological signature (and their relative functional ontologies) resulted *PRKCA* (angiogenesis), *EP300* (chromatin remodelling), *TP53* (apoptosis), *MAPK8* (gene expression), *PIK3R2* (cellular glucose omeostasis), *PPP3CB* (response to cytokines), *AKT1* (cell growth), *CASP3* (apoptosis), *SERPINE1* (angiogenesis), *MYC* (cell cycle),*CDK6* (cell cycle), *FAS* (apoptosis), *RPS6KA1* (response to stress), *RAC1* (cytoskeleton organization), *MAPK1* (differentiation), *CCND2* (cell cycle), *JUN* (immune response) and *CCND1* (cell cycle). We conducted a further supportive analysis by building a protein-protein interaction network this time based on target genes from the aforementioned GSEA gene list, therefore more restricted. The involved pathways largely overlapped with outomes from validated target network analysis (Supplementary Table 8).

## Discussion

In this study, we performed a comprehensive analysis of miRNAs as biomarkers of clinical behaviour of OC in patients receiving their first systemic chemotherapy in the neo−/adjuvant or metastatic setting. We also assessed the association between biological aspects of OC and the expression of specific miRNA sets. All our results were tested for external validation.

A total of 34 miRNAs expressed in the 60 IRE tumor samples were detected showing a significant impact on at least 1 among OS, EFS and PSS in the IRE patients’ cohort.

The 2 miRNAs whose expression was associated with longer OS in the IRE cohort, namely miR-1249-3p, and miR-3195, were not reported to impact OC in previous studies. However, they were associated with longer OS (miR-3195) and increased chemiosensitivity (miR-1249-3p) in a previous study of non small cell lung cancer (NSCLC) patients [18].

Previous data on part of the 17 miRNAs which impacted EFS in the IRE cohort often showed contrasting conclusions with respect to our results. In particular, concerning miRNAs associated with longer EFS in the IRE cohort, previous studies on miR-665 showed its association with poor prognosis in NSCLC and in breast cancer (BC) [19, 20]. Regarding miRNAs predicting a shorter EFS in the IRE cohort, earlier studies showed that at tissue level miR-101-3p was T/N-upregulated [21], as opposed to what we obsverved in our cohort (Supplementary Table 2), whereas miR-148-3p was T/N-downregulated, consistently to the IRE cohort (Supplementary Table 2). Lastly, in a previous study, miR-148 was associated with positive prognostic effect in OC patients, while negative prognostic effect was observed the IRE cohort [22].

Regarding the 3 miRNAs which resulted associated with both shorter EFS and P-res in the IRE cohort, findings from prior studies were mostly consistent with ours. In particular, studies found that both miR-224-5p [23] and miR-149-5p [24] were associated with P-res in OC patients.

Some interesting results from previous studies were also reported on some miRNAs among those 12 determining only PSS in the IRE cohort. In particular, among miRNAs predicting P-sens, miR-520e demonstrated an association with negative prognosis in a previous study of NSLC patients [25], therefore showing an opposite effect compared to our results. Concerning miRNAs predictive of P-res in the IRE cohort, the available evidence regarding other types of tumors is extensively concordant with our findings, even though no studies on OC were identified. Notably, MiR-33b-3p, miR-629-3p, and miR-301a, were respectively predictive of platinum resistance in NSCLC [26], and head and neck cancer (HNC) [27], and chemoresistance in pancreatic cancer (PaC) [28]. Moreover, association with shorter OS was reported for miR-629-3p in HNC [27] and NSCLC [29], and for miR-301a-3p in BC, in which it also determined shorter PFS [30].

The IRE cohort was heterogeneos in terms of disease stages and, consequently, patients’prognosis. In fact, the disease setting to which patients belonged, namely, neo-/adjuvant or metastatic, was associated with distinctive distributions of EFS, OS and PSS among the 3 subsets. However, miRNAs differentially expressed between P-res and P-sens patients, and miRNAs that resulted T/N-deregulated, could not yield a separation between these two groups when used in HCA (Fig. 2, Panel d), and in PCA (Fig. 3, Panel b), respectively. Moreover, miRNAs impacting OS and miRNAs impacting EFS and/or PSS in the IRE cohort seemed not to intersect with each other. Three miRNAs (miR-224-5p, miR-15b-5p, and miR-149-5p) were represented both among those impacting EFS and those impacting PSS, but this was expected, considering the strong relation between these two outcomes.

Furthermore, internal analysis in the IRE cohort demonstrated that only 3 of the 34 miRNAs with prognostic/predictive effect were also found among those with differential expression between the neoadjuvant, adjuvant, and metastatic setting. Specifically, miR-99a-5p, and miR-101-3p, associated with shorter EFS, and miR-500a-3p, predictive of P-res, resulted all upregulated in neo-adjuvant patients (Stage IIIC), with respect to adjuvant patients (Stage I - IIIB).

Finally, differential miRNA expression based on BRCA 1/2 mutational status showed only miR-512 dowregulated in mutated patients. This miRNA was not prognostic neither predictive in the IRE cohort. However, it demostrated tumor suppressive and chemotherapy sensitizing effects in previous studies of BC [31]. Overall, results from this subset of analysis show that differential miRNA expression in the IRE cohort is only partly determined by patients’ heterogeneity, while it is mostly a function of inter-tumor differences. This conclusion confers internal consistency to the analysis by which we detected 34 miRNAs with prognostic/predictive value inside the IRE cohort itself. Nevertheless, external consistency of our findings relatively to the impact of these miRNAs on clinical outcomes such as OS, EFS and PSS could not be confirmed when we tried to validate their prognostic/ predictive potential in the dataset based on miRNA sequencing of the TCGA cohort. This result might be partially reconciled with the possible insufficient matching in terms of patient and disease characteristics between the IRE and TCGA cohorts, difference in surgical and systemic treatment in the two cohorts, or even related to the different technologies used for miRNA profiling. For instance, the percentages of patients with disease in stage I to IIIB, IIIC and IV were respectively 18.3, 45 and 36.7% in the IRE cohort, and 11, 73 and 16% in the TCGA cohort. Regarding surgery, around 80% of IRE cohort patients and 90% of TCGA cohort patients underwent maximum cytoreduction. All patients in the IRE cohort received platinum-based systemic treatment which consisted in carboplatin plus paclitaxel in 90% of cases, and was delivered with a neo-/adjuvant, or palliative intent, according the disease stage. Similarly, nearly all patients in the TCGA cohort were treated with platinum-based systemic therapy, but detailed information on treatment protocols is not available, therefore making these two cohorts not fully comparable. The intra-cohort heterogeneity with regards to disease stage and treatment, and the inter-cohort imbalances for the same characteristics, weaken the possibility for a robust analysis of clinical outcomes in connection with  the biological aspects of the disease, and impairs the validation of possible biological-to-clinical implications between the IRE cohort and the TCGA cohort. On the other hand, due to the small sample size of the IRE cohort, it was not possible to conduct a more refined analysis by selecting groups stratified by stage from the IRE and TCGA cohorts, respectively. However, we hypothesized that an investigation mode rooted on an exclusively biological basis could offer more opportunities and help partially overcome such limits. This was the main reason why we explored T/N-deregulation of miRNAs in the IRE cohort and tried to validate it against the TCGA data as a first step towards identifying a biological signature. About 20.3% of the miRNAs differentially expressed between tumor tissues and control tissues in the IRE cohort were validated for such deregulation in the TCGA cohort. Moreover, in an additional experiment using RT-PCR, we selected a sample of 3 commonly deregulated miRNAs (miR-99a-5p, miR-145-5p, miR-224-5p), and quantified their expression in a subset of the IRE cohort, by confirming the same T/N-deregulation as detected by array profiling. Finally, due to the low number of normal samples in the TCGA cohort (8 samples), we used two additional datasets from the GEO, namely GSE119055 (6 ovarian cancer tissue samples and 3 normal ovarian tissue samples), and GSE83693 (8 primary ovarian cancer tissue and 4 normal ovarian tissue), to test the T/N-deregulation state of the 54 commonly T/N-deregulated miRNAs between the IRE and TCGA cohorts. Results showed that around 80% of them were confirmed with the same T/N-deregulation. Differential expression of miRNAs between tumor and normal tissue is crucial for their functional assessment. It was demonstrated by various studies that it is a global tendency for tumors to have an overall low miRNA expression, because of impared miRNA biosynthesis [32]. Furthermore, a decreased expression of the endoribonuclease responsible for miRNA biogenesis (Dicer), was associated with worse clinical outcomes in OC patients, behaving as an independent prognostic factor [33]. Our findings are concordant with this aspect, since all 8 prognostic miRNAs which resulted T/N-deregulated in the IRE cohort (miR-148a-3p, miR-320d, miR-361-5p, miR-99a-5p, miR-320a, miR-101-3p, miR-500a-3p, miR-664a-3p) had a lower expression in the tumor, when compared to normal tissues. We worked exclusively on the 54 miRNAs validated in the TCGA with respect to their T/N-deregulation, in the process of identifying sets of miRNAs cogent for signature composition and functional analysis. Two miRNAs from the IRE cohort fully satisfied this filtering process. Specifically, miR-99a-5p, and miR-320a, resulted both associated with shorter EFS in the IRE cohort, and with decreased expression in tumor tissue in both IRE and TCGA cohorts. The signature including both miRNAs, maintained significance for EFS, and showed potentials also for OS prediction in the IRE cohort, even though these results were not confirmed in the TCGA cohort. A previous study showed that plasma levels of miR-99a-5p were elevated in OC patients and were associated with pro-tumorigenic effect [34]. Studies on other solid tumors showed that both miR-99a-5p, and miR-320a, were T/N-downregulated at tissue level and this deregulation determined poor prognosis [35–37]. Moreover, preclinical studies demonstrated a tumor suppressive effect of both these miRNAs at the tissue level for a wide spectrum of solid tumors [38–46]. Overall, our findings and data from literature support the idea for the validity of these two miRNAs to be used as a prognostic signature. Moreover, the T/N-downregulation of the signature could have an independent negative prognostic value which can be tested in future studies. Besides these 2 T/N-downregulated miRNAs, we also selected for further evaluation miR-224-5p, an IRE T/N-upregulated miRNA (statistically significan in RT-PCR), with concordant deregulation in the TCGA cohort. Based on the fact that it was predictive for both shorter EFS and P-res in the IRE cohort, and considering the concordant evidence from literature focused on its role in OC and other solid tumors, we believe that this miRNA is a valid candidate to be studied as a biomarker for OC not only at the tissue level, but also by measuring its plasma level in future studies. It was demonstrated that bioactive miRNAs can be released into the body fluids from cancer cells, where they result very stable, being resistant to RNase degradation [47]. A further study on OC showed that circulating miRNAs are crucial for intercellular communication and metastatic spread [48, 49].

Besides their utility as prognostic/predictive biomarkers, miR-99a-5p, miR-320a, and miR-224-5p showed features which make them relevant for pathway analysis. In order to extend the set of the selected 3 miRNAs in prospect of functional analysis, we used the same methodology to seek for a prognostic signature and predictive miRNAs in both datasets relative to the TCGA cohort. Hence, by using the 54 validated miRNAs for T/N-deregulation, we identified 5 of them that were downregulated, associated with longer EFS and OS (miR-150, miR-30d, miR-342, miR-424, and miR-502), and constituting a relative signature that conserved prognostic value in the TCGA. We identified also 1 upregulated miRNA which was predictive of P-res (miR-200a). Altogether, the final set of 9 miRNAs was used for functional and pathway enrichment analysis, as a biological signature for OC. A further confirmation of the differential expression of these 9 miRNAs between normal ovarian tissue and OC tissue was obtained by performing PCA on their anti-correlated target genes profiled in 88 normal tissue samples from the GTXe dataset and the TCGA RNAseq dataset (Supplementary Fig. 6).

Overall, the TCGA cohort together with the GEO and GTXe datasets were used as validation datasets for T/N-deregulation, and the TCGA dataset was futherly utilized as an external data supplement for the conceptualization of a viable 9 miRNA signature.

Results of gene enrichment analysis performed separately for T/N-upregulated and T/N-downregulated miRNA target genes, and construction of a miRNA-mRNA regulatory network demonstrated the validity of the 9 miRNA signature as a biological fingerprint of OC. The selected 9 miRNAs resulted involved in the main biological processes which are hallmarks in the pathogenesis of OC. In particular, the predicted targets of the T/N-downregulated 7 miRNAs set were oncogenic pathways such as EMT, AKT, mTOR, ATF2, which have been implicated in various aspects of OC pathogenesis, diagnosis and treatment [50–52]. The most relevant target of the 2 T/N-upregulated miRNAs resulted the cell cycle regulation and MAPK/ERK pathway, which has been targeted by novel therapeutical agents seeking to overcome cisplatin resistance in OC [53]. We then performed two types of network analyses based on interactions between the target genes of the 9 miRNAs selected as biological signature. The core network was built by using gene interactions of validated targets, and a second supportive network was constructed on the basis of protein-protein interactions using targets restricted also by target prediction and T/N-deregulation. Results were extensively overlapping, and most affected pathways resulted MTOR, AKT, MAPK, P53, and Wnt signaling pathways, shown to be pivotal in OC biology [50, 51, 53–55].

A collateral aspect that deserves further consideration when discussing our study results, and which is supported by previous literature on miRNAs in OC and other cancers, is related to some generalizable features concerning their biological function. In first place, miRNA’s T/N-deregulation direction didn’t show to have a universal effect on their oncogenic/tumor suppressive activity, neither could down- or up-regulation be used as a surrogate for the negative or positive prognostic effect. Hence, our work and previous evidence did not support the existence of a fixed dogma for the function of miRNAs in the oncological landscape, such as the hypothetical: T/N-upregulation ➔ oncogenic effect ➔ drug resistance ➔ poor survival, or the inverse. It is consequential to think that the role of miRNAs in biological systems should be studied in a context encompassing other crucial factors affecting their function, such as, the co-expression of long non coding RNAs, which might exert sponging effect on miRNAs, and a definition of the predominant driver pathways in a specific preclinical or clinical experimental platform. Most of the currently available results of miRNAs in OC pathogenesis were obtained from cell lines in gain-and loss-of function experiments, which cannot reflect the complexity and dynamicity of the biological processess in human organisms. Hence, another way to improve reliability of results in future preclinical studies could implicate the use of organoids, which can resemble histological and genetic characteristics of OC [56]. Lastly, the relevance of studying miRNAs in OC or other cancers is not limited only to an informative function, but extends also to their potential as future therapeutical targets, especially as modulators of chemoresistance [57].

## Conclusion

In conclusion, our study corroborates previous evidence on the relevance of miRNA expression in understanding the clinical course and various biological aspects of OC. We detected a set of miRNAs impacting main clinical outcomes in OC patients. Moreover, based on differential miRNA expression between tumor and normal ovarian tissue, a wider group of miRNAs was identified, which is highly involved in crucial biological pathways of OC. The next step will contemplate the extension of this study with the inclusion of a larger and more homogenous patient population, in order to reach more standardized results, which can be easily validated against external datasets. Indeed, T/N-deregulation was confirmed as a hallmark when conducting miRNA profiling studies and future investigations should put more efforts also on this aspect. And finally, the role of miRNAs in OC should be studied while taking into account additional factors affecting their function at a molecular level. Having all this in mind, it is clear that the potential utility of miRNAs in OC still needs to be leveraged adequately.

## Supplementary Information

**Additional file 1.**

**Additional file 2.**

**Additional file 3.**

**Additional file 4.**

**Additional file 5.**

**Additional file 6.**

**Additional file 7.**

**Additional file 8.**

**Additional file 9: Figure S1.** Box plots displaying 3 miRNAs (panels A, B, C) with statistically significant (Wilcoxon test, *p* < 0.05) differential expression between 10 tumor samples and 10 normal tissue samples from the IRE cohort, when expression levels were measured with RT-PCR.

**Additional file 10: Figure S2.** Kaplan-Meier curves illustrating the performance of the IRE prognostic signature in the TCGA cohort for EFS (A), and OS (B). Kaplan-Meier curves showing the impact of miR-224-5p on EFS in the IRE cohort (C). Differences between curves were evaluated by logrank test. Box plots illustrating the expression level of miR-224-5p in tumor samples (T) versus normal tissue samples (N), in the TCGA cohort (D), and in the IRE cohort (E). Box plots showing the expression levels of miR-200a in platinum resistant tumors (resist) versus platinum sensitive tumors (resist), in the TCGA (F). Differences in miRNAs expression were assessed by Student’s T-test. For all the comparisons, the level of statistical significance was *p* < 0.05.

**Additional file 11: Figure S3.** Kaplan-Meier curves relative to the 5 miRNAs of the TCGA prognostic signature, showing their impact on EFS. Statistical significance was established by logrank test. For all the comparisons, the level of statistical significance was *p* < 0.05.

**Additional file 12: Figure S4.** Kaplan-Meier curves relative to the 5 miRNAs of the TCGA prognostic signature, showing their impact on EFS. Statistical significance was established by logrank test. For all the comparisons, the level of statistical significance was *p* < 0.05.

**Additional file 13: Figure S5.** Volcano plot illustrating differential miRNA expression between tumoral and normal tissues in the IRE cohort, with labels on the 9 miRNAs selected for the biological signature (A). Box plots showing the expression levels comparison of the 9 miRNAs included in the biological signature, in the tumoral tissue (T) versus normal tissue (N), in the IRE cohort (B), and in the TCGA cohort (C). Statistical significance was assessed by permutation test and Student’s T-test.

**Additional file 14: Figure S6.** Principal component analysis of 88 normal samples from GTEx dataset and 499 tumor samples from TCGA RNA sequencing-based dataset, using all the target genes negatively correlated to the 9 miRNA signature and significantly modulated between normal samples and tumor samples.

## Data Availability

The datasets used and/or analysed during the current study are available from the corresponding author on reasonable request.

## Abbreviations

OC: Ovarian cancer
EMT: Epithelial-mesenchymal transition
CSC: Cancer stem cells
miRNA: MicroRNA
IRE: Regina Elena National Cancer Institute
TCGA: Cancer Genome Atlas
OS: Overall survival
EFS: Event free survival
PSS: Platinum sensitivity state
P-res: Platinum resistant
P-sens: Platinum sensitive
HCA: Hierarchical clustering analysis
PCA: Principal component analysis
