# Supplementary figures and images for: MicroRNA-based signatures impacting clinical course and biology of ovarian cancer: a miRNOmics study
Source: Biomark Res. 2021 Jul 13;9:57. doi: 10.1186/s40364-021-00289-6 (PMC8276429; doi:10.1186/s40364-021-00289-6)

**A**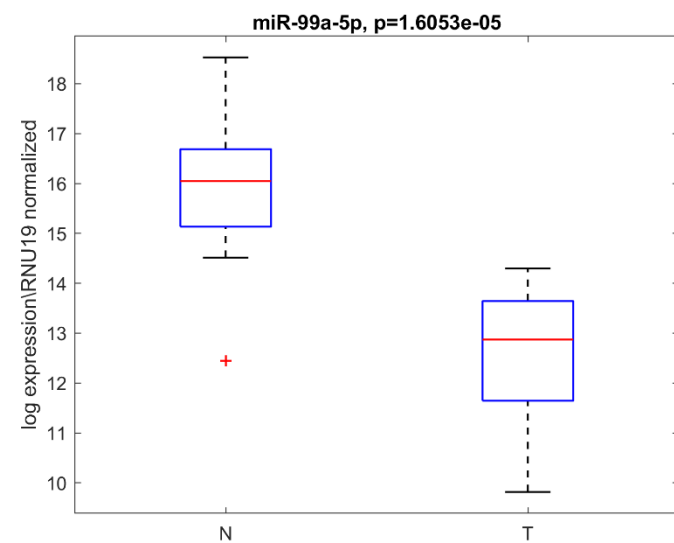**B**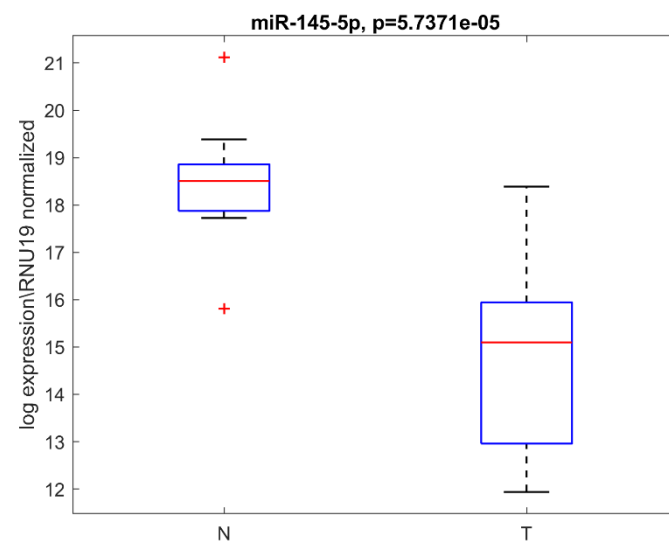**C**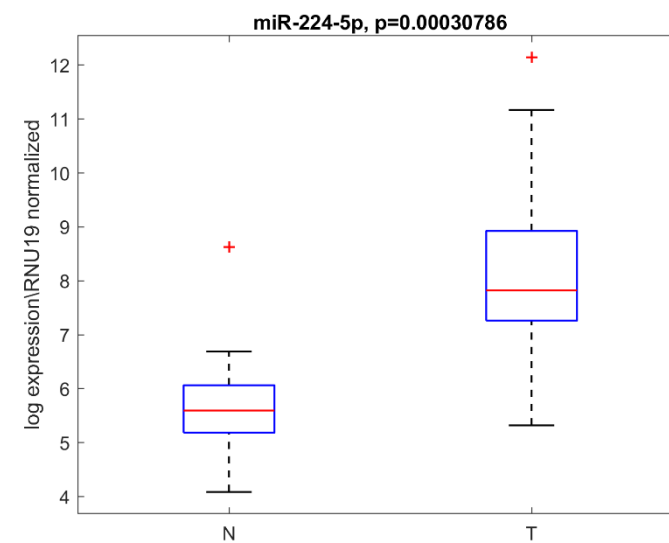

Supplement: Supplementary file 9 — Additional file 9: Figure S1. Box plots displaying 3 miRNAs (panels A, B, C) with statistically significant (Wilcoxon test, p < 0.05) differential expression between 10 tumor samples and 10 normal tissue samples from the IRE cohort, when expression levels were measured with RT-PCR. [file 40364_2021_289_MOESM9_ESM.pdf]

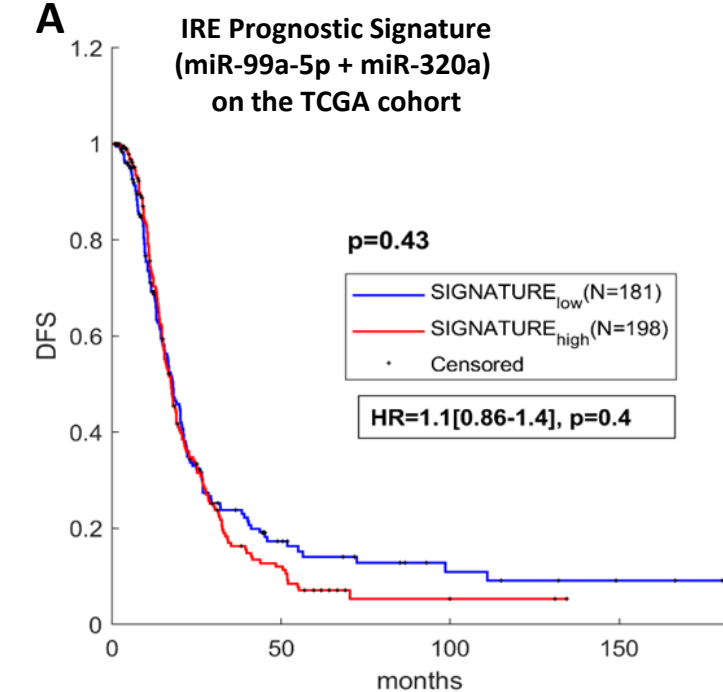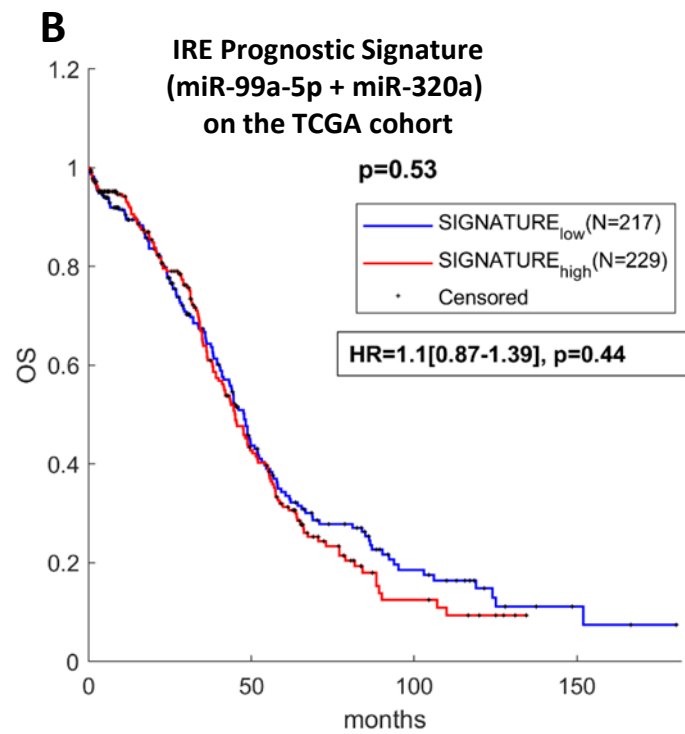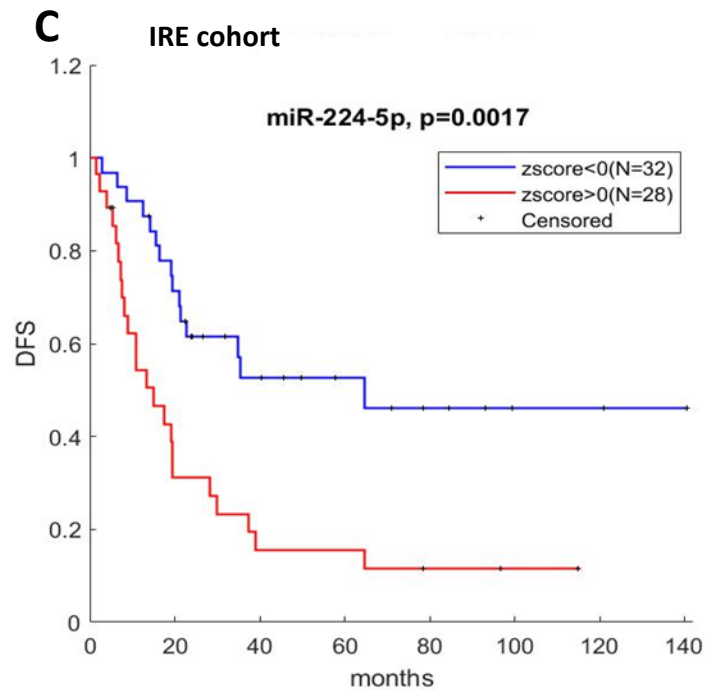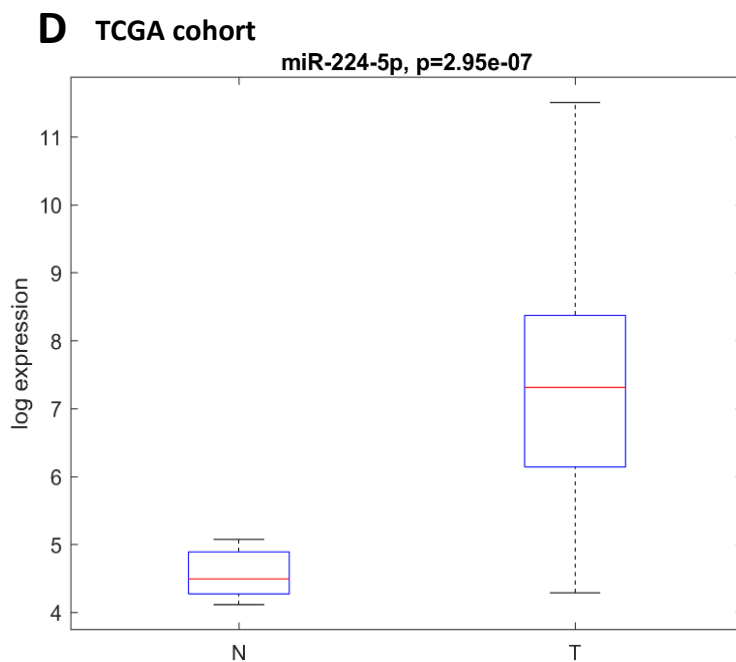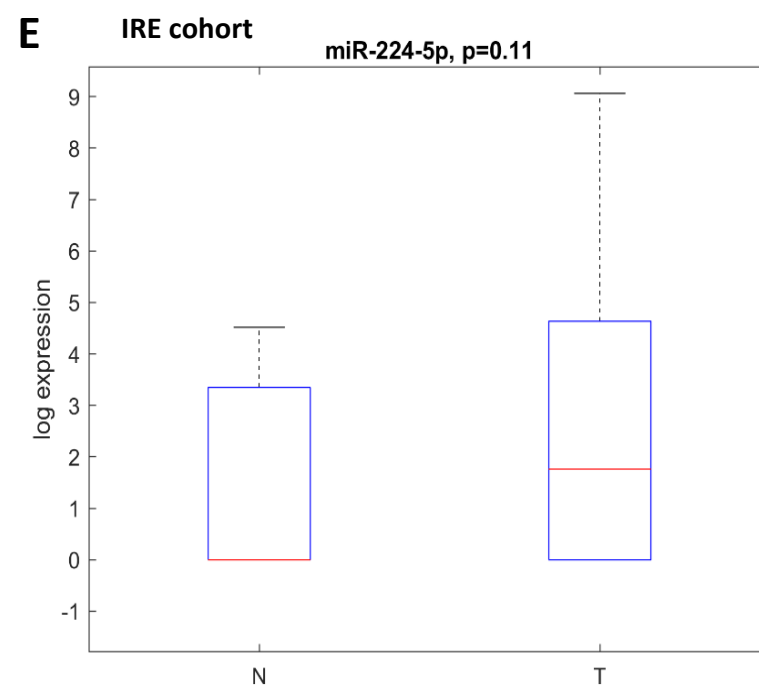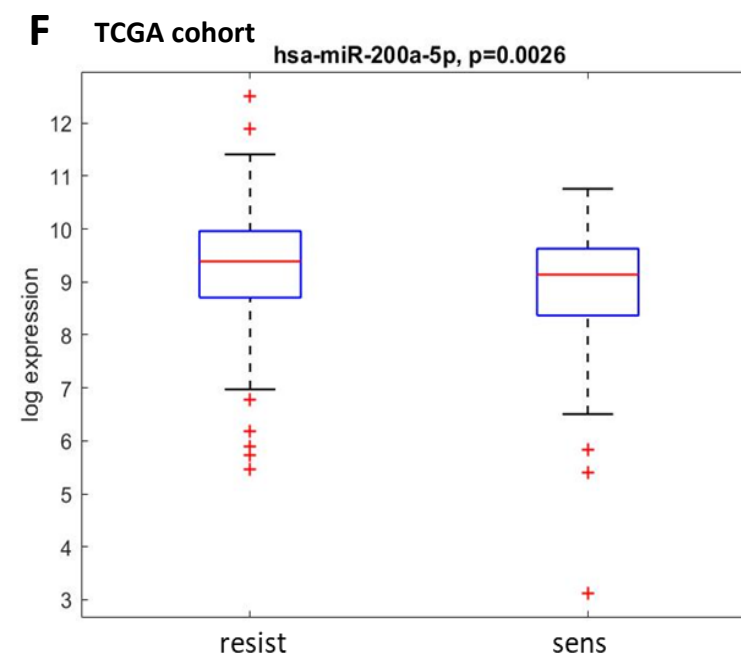

Supplement: Supplementary file 10 — Additional file 10: Figure S2. Kaplan-Meier curves illustrating the performance of the IRE prognostic signature in the TCGA cohort for EFS (A), and OS (B). Kaplan-Meier curves showing the impact of miR-224-5p on EFS in the IRE cohort (C). Differences between curves were evaluated by logrank test. Box plots illustrating the expression level of miR-224-5p in tumor samples (T) versus normal tissue samples (N), in the TCGA cohort (D), and in the IRE cohort (E). Box plots showing the expression levels of miR-200a in platinum resistant tumors (resist) versus platinum sensitive tumors (resist), in the TCGA (F). Differences in miRNAs expression were assessed by Student’s T-test. For all the comparisons, the level of statistical significance was p < 0.05. [file 40364_2021_289_MOESM10_ESM.pdf]

**A**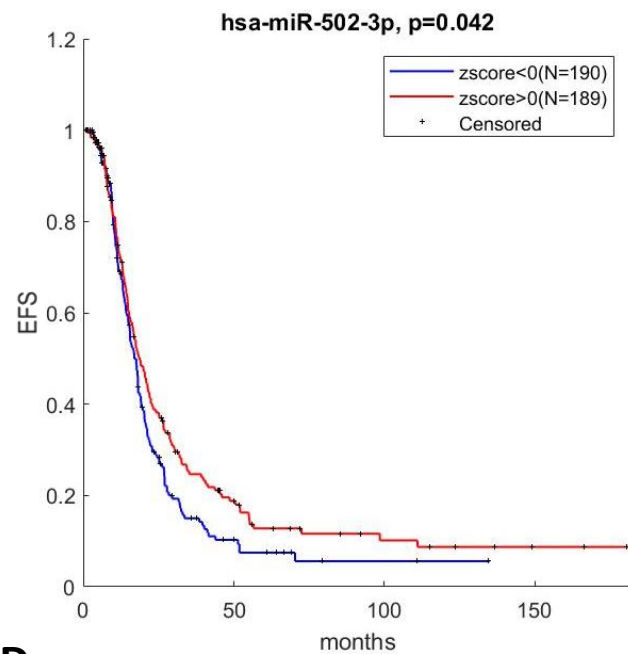**B**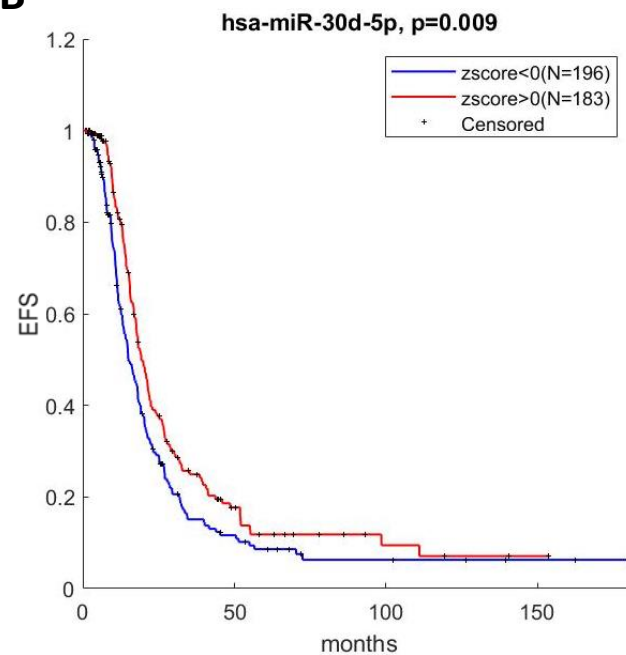**C**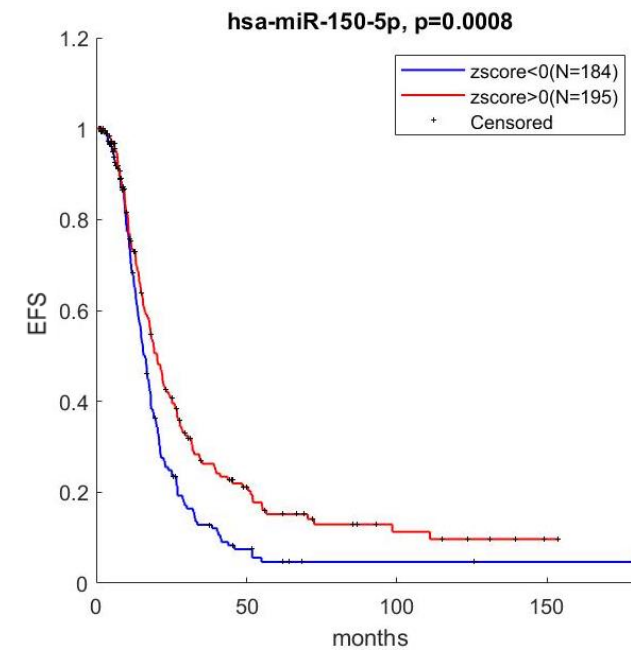**D**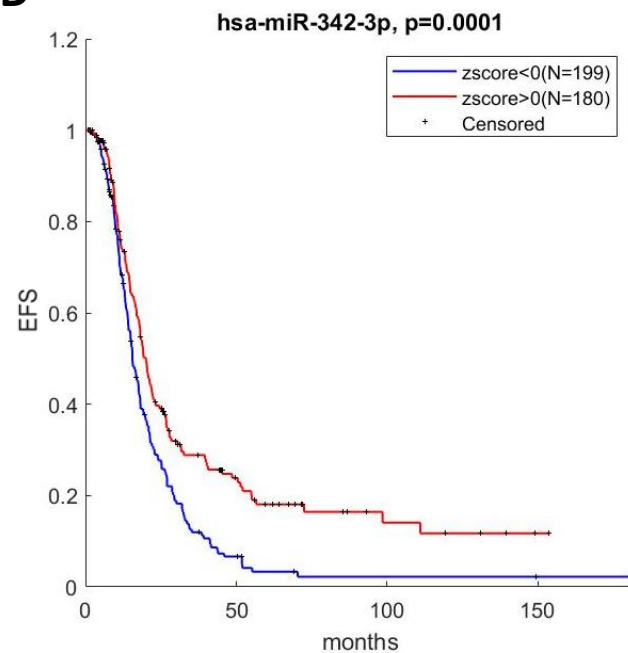**E**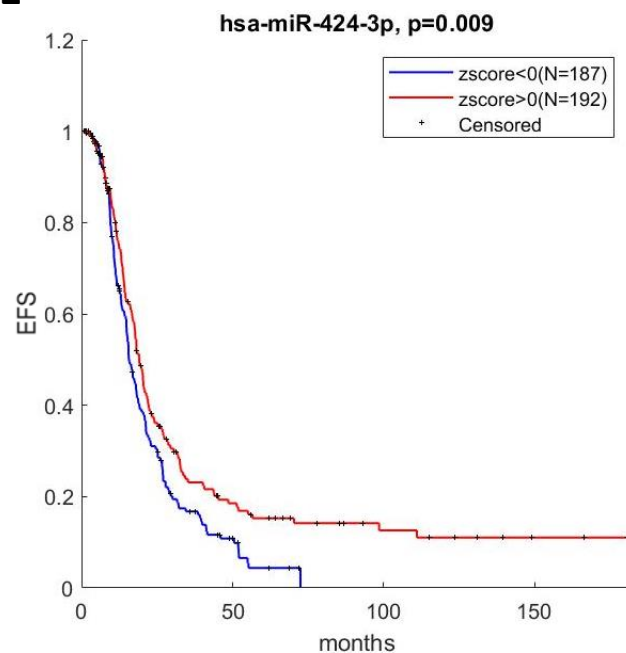

Supplement: Supplementary file 11 — Additional file 11: Figure S3. Kaplan-Meier curves relative to the 5 miRNAs of the TCGA prognostic signature, showing their impact on EFS. Statistical significance was established by logrank test. For all the comparisons, the level of statistical significance was p < 0.05. [file 40364_2021_289_MOESM11_ESM.pdf]

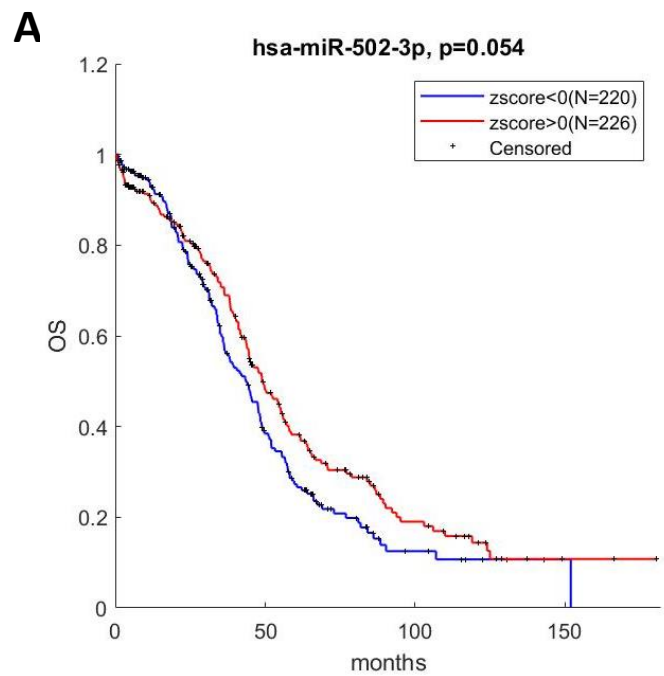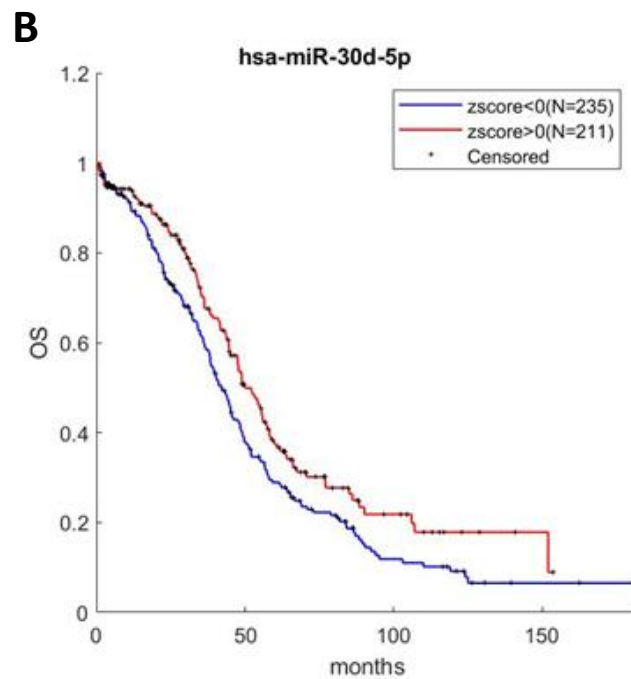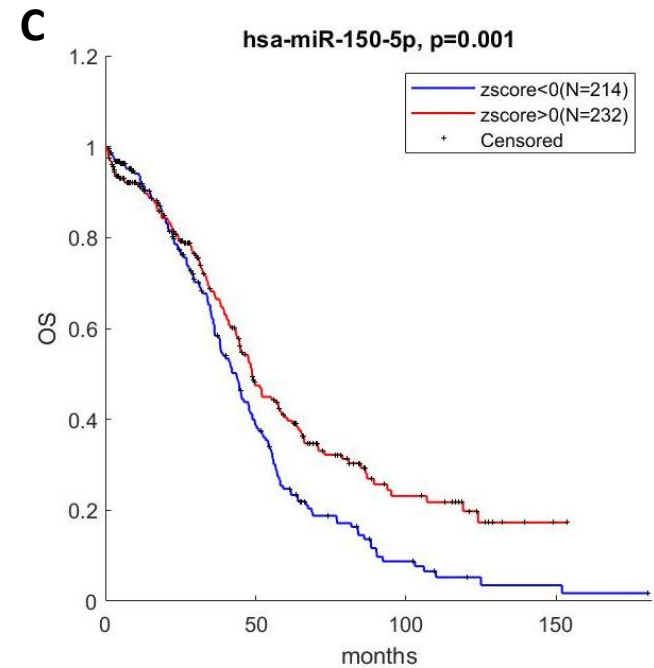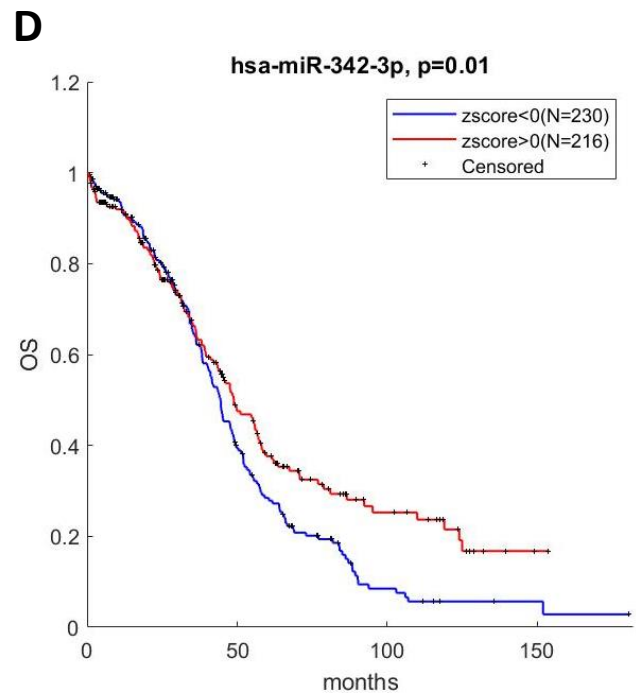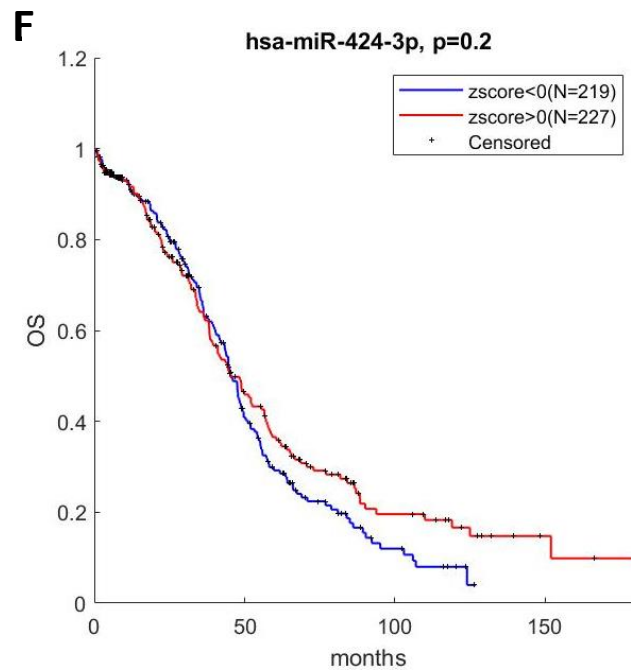

Supplement: Supplementary file 12 — Additional file 12: Figure S4. Kaplan-Meier curves relative to the 5 miRNAs of the TCGA prognostic signature, showing their impact on EFS. Statistical significance was established by logrank test. For all the comparisons, the level of statistical significance was p < 0.05. [file 40364_2021_289_MOESM12_ESM.pdf]

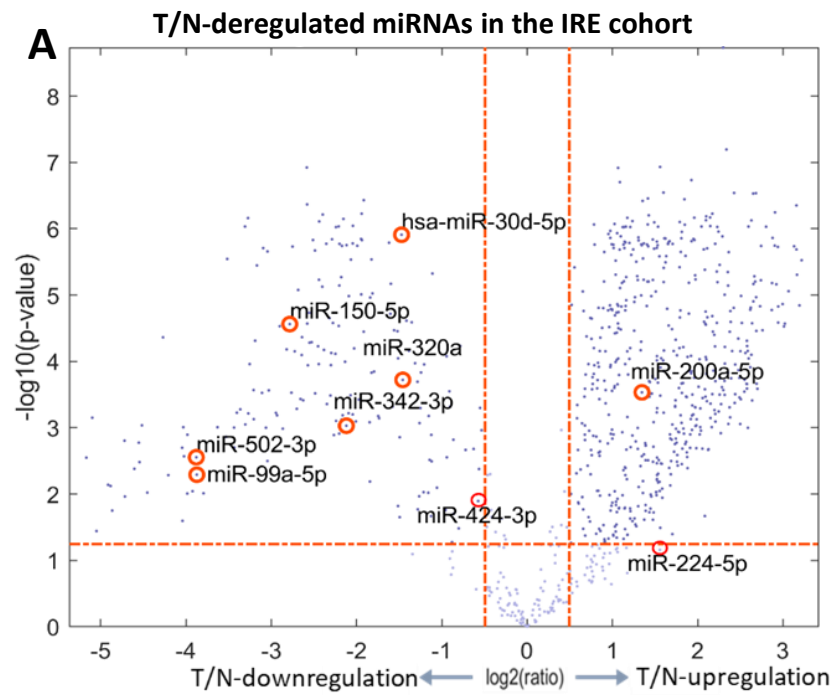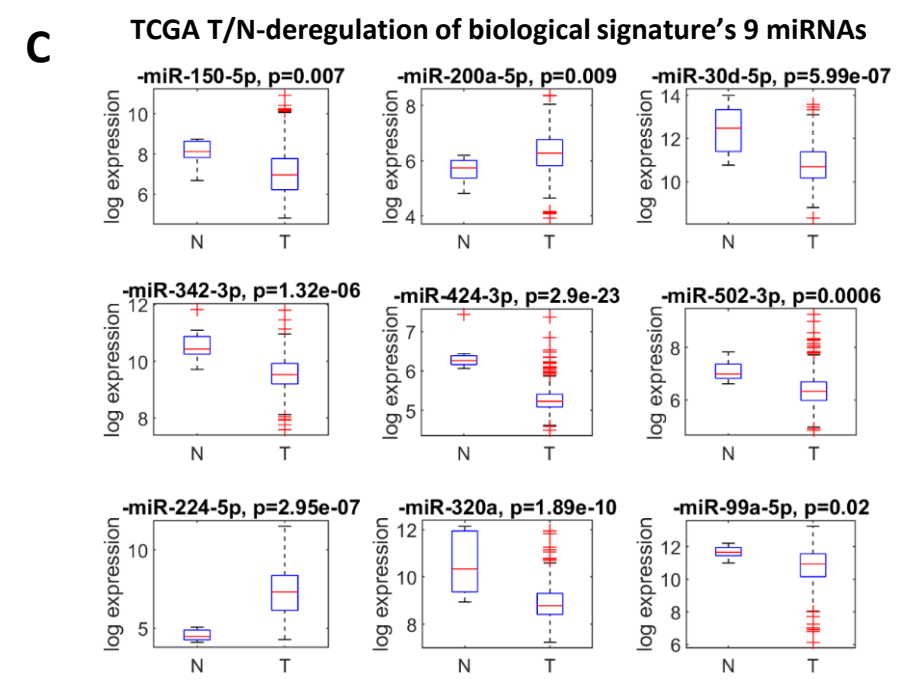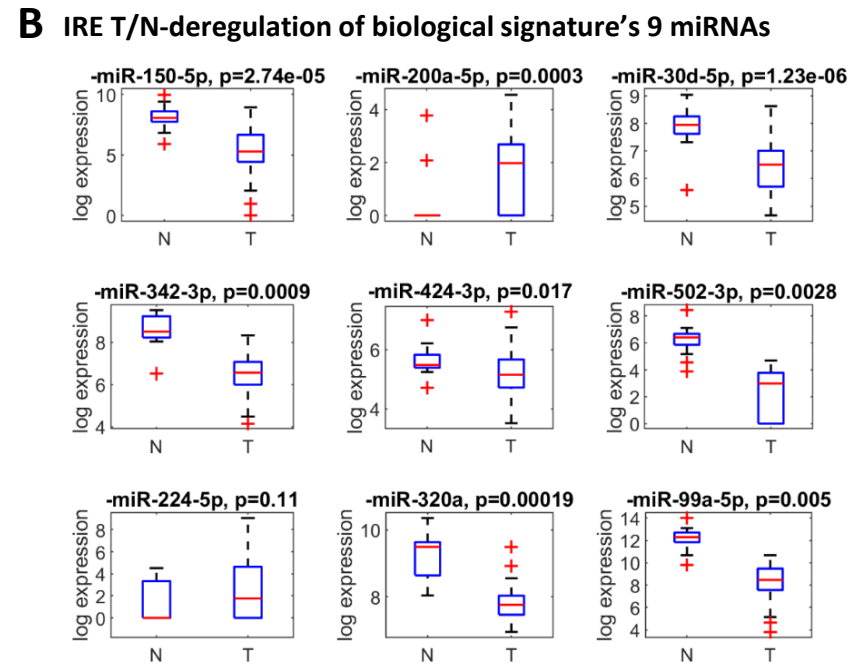

Supplement: Supplementary file 13 — Additional file 13: Figure S5. Volcano plot illustrating differential miRNA expression between tumoral and normal tissues in the IRE cohort, with labels on the 9 miRNAs selected for the biological signature (A). Box plots showing the expression levels comparison of the 9 miRNAs included in the biological signature, in the tumoral tissue (T) versus normal tissue (N), in the IRE cohort (B), and in the TCGA cohort (C). Statistical significance was assessed by permutation test and Student’s T-test. [file 40364_2021_289_MOESM13_ESM.pdf]

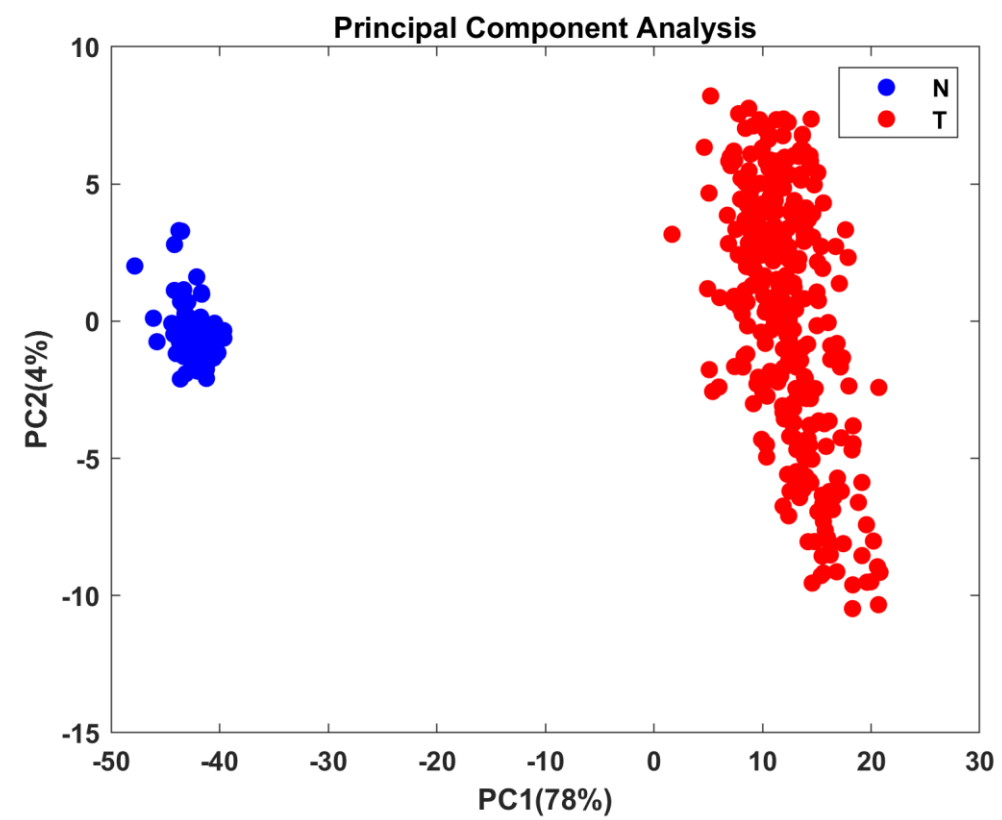

Supplement: Supplementary file 14 — Additional file 14: Figure S6. Principal component analysis of 88 normal samples from GTEx dataset and 499 tumor samples from TCGA RNA sequencing-based dataset, using all the target genes negatively correlated to the 9 miRNA signature and significantly modulated between normal samples and tumor samples. [file 40364_2021_289_MOESM14_ESM.pdf]
